# Validation of a commercial version of a competitive enzyme linked immunosorbent assay for the detection of antibodies to *Besnoitia besnoiti*

**DOI:** 10.1186/s13071-022-05591-2

**Published:** 2022-12-06

**Authors:** Gereon Schares, Andrea Bärwald, Marie-Astrid Vernet, Frédéric Bernard, Béatrice Blanchard, Philippe Coppe

**Affiliations:** 1 Institute of Epidemiology, Friedrich-Loeffler-Institut, Federal Research Institute for Animal Health, Insel Riems, Südufer 10, 17493 Greifswald, Germany; 2 Bio-X Diagnostics, 38 Rue de La Calestienne, 5580 Rochefort, Belgium

**Keywords:** Bovine besnoitiosis, cELISA, Cross-reactivity, Inhibition ELISA, Serological test

## Abstract

**Background:**

Several reports suggest a further spread of besnoitiosis to countries in which *Besnoitia besnoiti*-infected bovine herds have not been noticed yet. Cattle infected without clinical signs may represent reservoirs. Serological analyses in affected herds or animals from endemic regions are necessary to identify subclinical or inapparent infections and stop transmission to naïve animals or herds. The Monoscreen AbELISA *Besnoitia besnoiti* (BIO K 466) is based on a previously published in-house competitive ELISA, the Bb-cELISA1, but has a different test architecture. The present study aimed to use sera from a previous evaluation of Bb-cELISA1 to assess whether BIO K466 shows identical results. In addition, further well-characterized positive and negative samples were analysed to estimate diagnostic sensitivity and specificity.

**Methods:**

A first set of sera consisted of a total of 305 bovine sera, collected from German herds infected by *B. besnoiti*, *Neospora caninum* or *Sarcocystis* spp. Sera had been characterized by reference serological tests (i.e. immunoblot, immunofluorescence antibody test and an in-house indirect ELISA). A second set consisted of 200 confirmed *B. besnoiti*-positive sera from French herds. Negative cattle sera (*n* = 624) originated from Norway and The Netherlands, countries in which bovine besnoitiosis has not been reported yet.

**Results:**

Using the first set of sera, the BIO K466 showed an estimated diagnostic sensitivity of 97.9% (95% CI: 91.9%–99.6) and a diagnostic specificity of 99.5% (95% CI: 96.9%–100%) relative to reference serological tests. A direct comparison of the results revealed an almost perfect agreement between the results of the in-house Bb-cELISA1 and the commercialized version (kappa 0.98; 95% CI: 0.95–1). The validation using positive bovine sera from France and negative sera from other European countries revealed a diagnostic sensitivity of 97.5% (95% CI: 93.9%–99.1%) and specificity of 99.5% (95% CI: 98.5%–99.9%).

**Conclusion:**

In conclusion, BIO K 466 appears to be a suitable tool to diagnose bovine besnoitiosis, but needs further validation especially in cases of inconclusive, suspected false-positive or -negative results in other serological tests.

**Graphic abstract:**

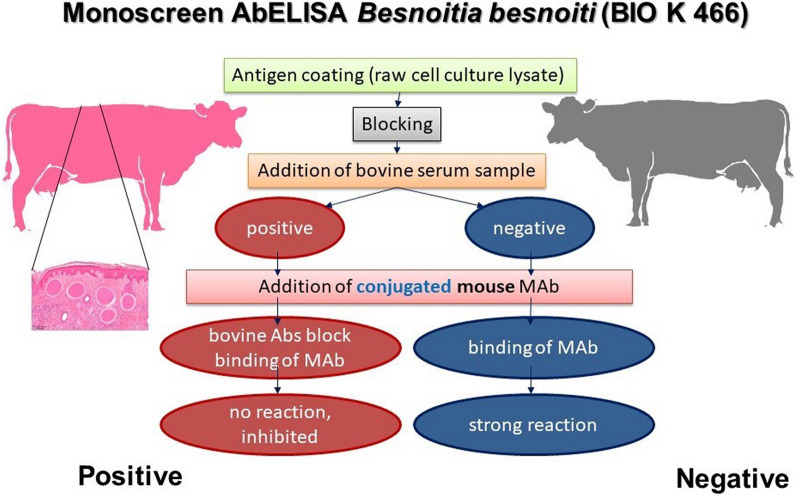

**Supplementary Information:**

The online version contains supplementary material available at 10.1186/s13071-022-05591-2.

## Background

Bovine besnoitiosis represents a problem in cattle farming and is of growing importance in some countries in Europe, including France, Spain and Italy [1, 2] and also Belgium, where many cases of infected herds were recently reported [3]. Bovine besnoitiosis is caused by *Besnoitia besnoiti*, a single-celled protozoon, closely related to other parasitic pathogens, such as *Toxoplasma gondii*, *Neospora caninum* and *Sarcocystis* spp. In contrast to the other parasites, for which carnivores represent definitive hosts, able to shed environmentally resistant oocysts or sporocysts via faeces into the environment, definitive hosts remain unknown for *B. besnoiti* [4]. The only established mode of infection of cattle is horizontal via biting and blood-sucking insects, such as tabanids and *Stomoxys* spp. [5–7]. Insects are able to transfer the infection by horizontal mechanical transmission, but most likely only over very short distances [8]. Animal trade and transportation as well as the inadvertently incorporation of infected animals into naïve herds are regarded the main way by which the infection spreads in regions and crosses borders of countries [1, 2] as recently also mentioned in an expert elicitation [9].

Thus, serological testing of animals prior to incorporation into another herd is efficient to prevent accidental restocking a naïve herd with infected animals [1, 2]. The only limitation is that there is only a short window during acute infection in which animals are not yet serological positive but probably able to transmit infection [10, 11]. During the early course of infection, clinical signs may prevent transport of animals but the signs of besnoitiosis are not always so severe that they are noticed by farmers [8].

Serological differences in their diagnostic characteristics and especially insufficiencies in the diagnostic specificity of serological tests including commercially available tests may cause inconclusive results or results suspected of being false positive [2, 12, 13]. Inconclusive or test results suspected to be false-positive cause additional costs, because the serological status of an animal needs to be determined using non-standardised serological tests, such as immunoblot (serological testing of a Western blot), usually only available in specialized laboratories. In addition, suspected cases might only be clarified after an intensive clinical inspection as well as by molecular analyses to confirm or to discard the hypothesis of an existing infection.

To establish a serological test with high diagnostic specificity, a first competitive enzyme-linked immunosorbent assay (Bb-cELISA1) had been settled [11]. This in-house test was based on a mouse monoclonal antibody (Mab 1/24-9-1A4) recognizing an epitope on the surface of *B. besnoiti* tachyzoites. This Mab was also used for the establishment of a commercial cELISA, the Monoscreen AbELISA *Besnoitia besnoiti* (BIO K 466). Although using the same Mab, the test designs differ between the original in-house and the commercial version of this competitive ELISA. Because there is no published information on the suitability of this new commercial test yet, the aim of the present study was to use sera from the previous evaluation of the in-house Bb-cELISA1 to study whether the novel test shows identical results or not. In addition, diagnostic characteristics were assessed by an additional set of sera including samples from infected French cattle and negative cattle from other European countries not known to have experienced cases of besnoitiosis. Results suggest both high diagnostic sensitivity and specificity of the new test.

## Methods

### Sera

For this evaluation two sets of bovine sera were used. A first set consisted of a total of 305 bovine sera, collected from two German herds infected by *B. besnoiti*, *Neospora caninum* or *Sarcocystis* spp. These sera represented a subset of those sera which had been used to validate the in-house Bb-cELISA1 [11]. The *B. besnoiti*-positive sera had been characterized by four reference serological tests (i.e. two immunoblots, an immunofluorescence antibody test [IFAT] and the in-house BbAPureELISA). The selected positive sera were positive in at least two of the reference tests and represented different levels of positivity in IFAT (Table 1).

**Table 1:** Bovine sera selected for the present study from a serum panel previously used to validate the in-house Bb-cELISA1 [11]

Herd (number of herds)	Tests used to characterize sera	Status	Number (additional information)	Number of sera used for estimates of diagnostic sensitivity and specificity (reference)
Besnoitiosis herds (*n* = 2)	*B. besnoiti* tachyzoite IFAT, tachyzoite immunoblot, bradyzoite immunoblot, BbAPureELISA and Bb-cELISA1	*Besnoitia* positive	95 (positive IFAT titre, 1:100 [*n* = 19], 1:200 [*n* = 20], 1:400 [*n* = 18], 1:800 [*n* = 10], 1:1600 [*n* = 10], 1:3200 [*n* = 10], 1:6400 [*n* = 4])	95 (reference positive)
		*Besnoitia* negative	9 (positive IFAT titre, 1:100 [*n* = 7], 1:200 [*n* = 1])	8 (reference inconclusive), 1 (reference negative)
Neosporosis herd (*n* = 1)	*N. caninum* p38 ELISA, *N. caninum* p38 avidity ELISA	*Neospora* positive	97 (including 6 with abortion)	97 (reference negative)
		*Neospora* negative	4	4 (reference negative)
Sarcocystosis herd (*n* = 1)	Serologically not confirmed	Sera from suspected *Sarcocystis* spp. infections^a^	100	100 (reference negative)

*Besnoitia besnoiti* antibodies in herds with besnoitiosis were assessed using *B. besnoiti* tachyzoite immunofluorescence antibody test (IFAT) and IFAT seropositivity (reciprocal titre ≥ 100) confirmed by *B. besnoiti* tachyzoite immunoblot, bradyzoite immunoblot and BbAPureELISA [11]. Seropositivity for *N. caninum* was determined by p38 ELISA and sera further characterized by p38 avidity ELISA (Additional file 1: Dataset S1)

^a^Although the *Sarcocystis* spp. status of individual animals was unknown, frequent cases of eosinophilic myositis were recorded for this herd at slaughter

A second set consisted of 824 bovine sera including 200 sera positive for *B. besnoiti* from 12 French herds from 10 different French departments (Ariège, Aude, Tarn, Aveyron, Lozère, Ardèche, Drôme, Isère, Haute-Savoie, Allier). The animals, which had tested positive in immunoblot [14], were provided by the French National Reference Laboratory for Besnoitiosis, Maisons-Alfort, for the official validation of this test in France. The remaining 624 sera were regarded as likely *B. besnoiti* negative and collected in Norway (*n* = 260) and The Netherlands (*n* = 364) where bovine besnoitiosis has not been reported yet.

### Serological tests

Tests used to characterize sera for serological positivity against *B. besnoiti* or *N. caninum* (*B. besnoiti*: IFAT, tachyzoite immunoblot, bradyzoite immunoblot [15], BbAPureELISA [10], Bb-cELISA1 [11]; *N. caninum*: p38 ELISA, p38 avidity ELISA; Table 1) have been described in a previous study [11]. The commercial test under validation, the monoscreen AbELISA Besnoitia besnoiti, BIO K 466 (Bio-X Diagnostics, Rochefort, Belgium) is based on the same monoclonal antibody (1/24-9-1A4) reported previously, recognizing an epitope on the surface of *B. besnoiti* tachyzoites, and was used to establish the in-house Bb-cELISA, previously [11]. In brief, BIO K 466 ELISA plates are coated by a lysate of *B. besnoiti* tachyzoites purified from cell culture and subsequently blocked.

All samples were tested using the BIO-X Diagnostics kit strictly according to the manufacturer’s instructions. After adding 50 µl of a dilution solution to each well, 50 µl of serum samples is loaded and plates incubated for 2 h at 37 °C. After washing (3 × 300 µl, washing solution), 100 µl conjugate solution (i.e. Mab 1/24–9-1A4 coupled to peroxidase) is added to each well and incubated (30 min, 37 °C). After washing (3 × 300 µl, washing solution), 100 µl of a 3,3′, 5,5′-tetramethylbenzidine solution is added and colour development (21 °C, 10 min) stopped by adding 50 µl stopping solution (1 M phosphoric acid) per well. Optical densities are read out using a plate spectrophotometer with a 450-nm filter within 5 min after adding the stopping solution. Each plate contains wells in which a positive and negative control serum is tested; control serum results are used for validation (OD negative serum – OD positive serum > 0.700) and calculation of the percentage by which the test serum inhibits the reaction of the of the Mab 1/24-9-1A4 peroxidase conjugate (inhibition = (OD negative control serum – OD sample)/OD negative control serum). A sample is considered positive if its inhibition is > 0.50 (Table 2).

**Table 2:** Results in Monoscreen AbELISA *Besnoitia besnoiti* (BIO K 466), applying two sets of sera, i.e. a first set applied in a previous study and a second set established for present study

Sera used for validation	Result in Monoscreen AbELISA *Besnoitia besnoiti*	
	Positive	Negative
First set of sera (*n* = 305)		
Reference positive	93	2
Reference negative	1	201
Second set of sera (*n* = 824)		
Positive	195	5
Negative	3	621

### Statistical analyses

R, version 4.0.2 (R Foundation for Statistical Computing, Vienna, Austria; http://www.R-project.org) and the R package “optimal.cutpoints” were used to determine diagnostic sensitivity and specificity including 95% confidence intervals (95% CI). Correlation coefficient was calculated in R using the command “lm”. Kappa value was calculated using an online tool (http://vassarstats.net/kappa.html). Figures were assembled using R, version 4.0.2 (packages “ggplot2” and “scales”).

## Results and discussion

### Validation using sera from a previously study to characterize the in-house Bb-cELISA1

Using the first set of sera, previously established to characterize the in-house competitive ELISA Bb-cELISA1, the BIO K 466 showed a diagnostic sensitivity of 97.9% (95% CI: 91.9% – 99.6%) and diagnostic specificity of 99.5% (95% CI: 96.9% – 100%) relative to reference consisting of four previously published tests, i.e. two immunoblots using *B. besnoiti* tachyzoite or bradyzoite antigens [15], IFAT based on tachyzoite antigen [15] and an in-house BbAPureELISA [10] were similar to those of the in-house Bb-cELISA1 [11] (Additional file 1: Dataset S1).

A further validation, using 200 immunoblot-confirmed positive bovine sera from France and 628 negative sera from other European countries, where cases of bovine besnoitiosis have not reported yet (Additional file 2: Dataset S2), and revealed a diagnostic sensitivity of 97.5% (95% CI: 93.9%–99.1%) and specificity 99.5% (95% CI: 98.5%–99.9%).

So far, only indirect ELISA tests based on total tachyzoite cell lysates, lyophilized tachyzoites or semi-purified but nevertheless complex tachyzoite antigens (i.e. antigens enriched for diagnostically relevant antigens) have been established. This also includes a number of commercialized tests, and no competitive ELISA was available to diagnose besnoitiosis yet [2]. For these indirect ELISA tests, median diagnostic sensitivities of 92.2% (minimum 75.5%, maximum 100%) and median diagnostic specificities of 97.3% (minimum 93%, maximum 100%) were reported, as retrieved from previously reviewed publications [2]. Sets of sera used to validate test characteristics of various indirect ELISA were often not the same; in addition, criteria to define positivity vary between studies. Thus, estimates on diagnostic characteristics provided by different groups for these ELISAs are not directly comparable. However, in the present study, and independent of the set of sera used, levels for estimates for diagnostic sensitivity and specificity reached values exceeding those reported previously. This is an indication that the diagnostic characteristics for the BIO K 466 are probably better than at least for some of the indirect in-house and commercial *B. besnoiti* ELISAs based on crude or semi-purified antigens. However, because authors of this publication have competing interests, an independent validation by others using additional sets of reference sera, optimally testing some of other indirect ELISAs in parallel, is needed.

**Fig. 1:**
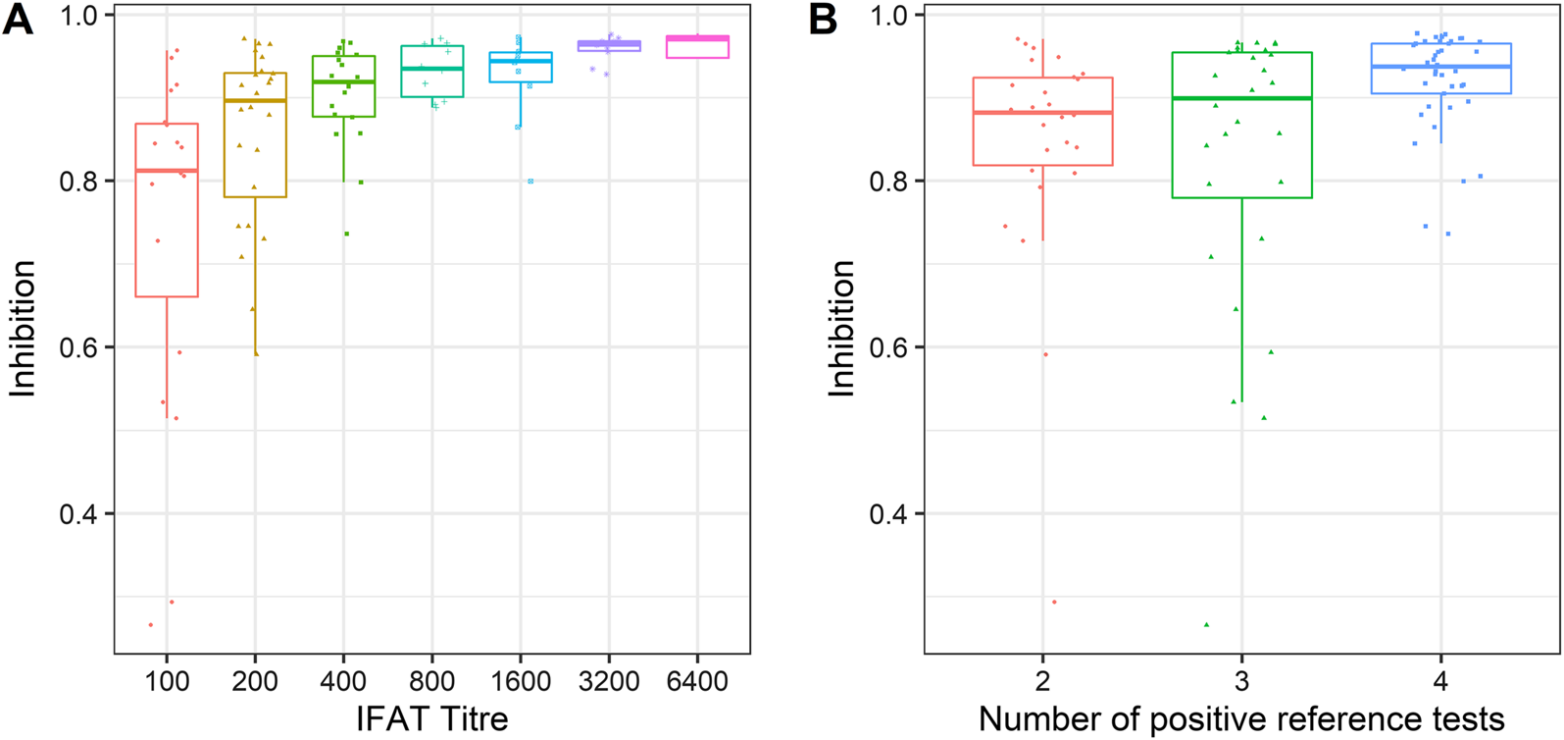
Comparison of the Monoscreen AbELISA *Besnoitia besnoiti* (BIO K 466) with reference tests using the first set of reference sera. **A** Relationship between the inhibition determined by BIO K 466 and the *B. besnoiti* IFAT titre in reference-positive samples. **B** Relationship between the inhibition determined by BIO K 466 and the number of positive results in *Besnoitia besnoiti* reference tests for individual reference-positive samples

One of the five tests used to characterize *B. besnoiti* positivity in the first set of sera (i.e. four reference tests and the Bb-cELISA1) used in this study was the IFAT. IFAT titres directly reflect the concentration of antibodies in a serum, specific for the surface of *B. besnoiti* tachyzoites. When selecting sera for this study we specifically focussed on sera which showed only low IFAT titres (1:100, 1:200) and added fewer sera with high or very high IFAT titres to the first panel. In the comparison of IFAT titres vs. the inhibition values in BIO K 466, all sera with IFAT titres of 1:200 and most (17/19) sera with IFAT titres of 1:100 showed inhibition in the competitive ELISA exceeding the cut-off of 0.50 (Fig. 1A). This finding is in accord with a previous study [15], which showed that at an IFAT titre of 1:100 the diagnostic specificity of this test started to fade and IFAT tended to produce false-positive results, at least under the test conditions in our laboratory [15].

The median values for the inhibition in BIO K 466 for *B. besnoiti*-positive bovine sera increased with the number of positive results which had been gained for individual sera in reference tests (Fig. 1B). The only two false-negative sera in BIO K 466 were positive in two or three of the four reference tests only (Fig. 1B). In addition, the results in Fig. 1B show that, even in cases where not all reference tests are positive, BIO K 466 was sensitive enough to test these sera positive.

### Relation of inhibition in the Monoscreen AbELISA *Besnoitia besnoiti* to those obtained with the in-house Bb-cELISA1

**Fig. 2:**
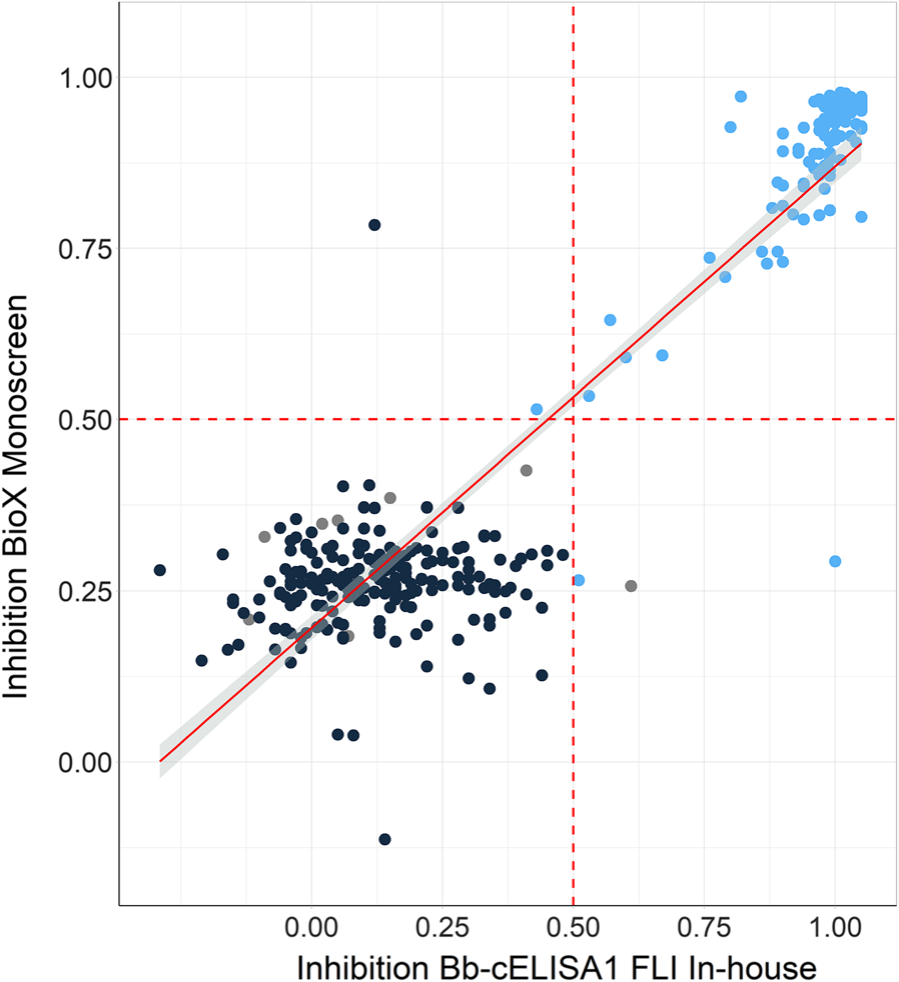
Correlation of the inhibition values obtained in the Monoscreen AbELISA *Besnoitia besnoiti* (BIO K 466) and the Bb-cELISA (FLI in-house) including *Besnoitia besnoiti* reference-positive (blue), reference-negative (black) samples and reference-negative samples but with positive immunofluorescence test titre (grey) and broken lines (red) represent cut-offs applied in the tests. The correlation as determined by linear regression was characterized by *R*^2^ = 0.86. The red line represents the regression line and the shaded area the 95% confidence limits

The results of the BIO K 466 with the results in the in-house Bb-cELISA1 were well correlated, as characterized by a relatively high *R*^2^ value of 0.86 (Fig. 2). In addition, the cut-offs applied in both tests seem to be very similar and the positive-negative test result of both tests largely agreed (Fig. 2) with only five sera of a total number of 305 showing divergent results. These five sera consisted of two which were reference-positive and tested false-negative in BIO K 466; one reference positive serum tested correct-positive in BIO K 466 but false-negative in the in-house Bb-cELISA1, a reference-negative serum tested false positive in the BIO K 466 and the remaining serum was reference-negative but with an immunofluorescence titer of 1:100 which tested correct-negative in BIO K 466 but false-positive in the in-house Bb-cELISA1 (Fig. 2).

Overall, a direct comparison of the results revealed an almost perfect agreement between the results of the in-house Bb-cELISA1 and the commercial version (kappa 0.98; 95% CI: 0.95–1). Thus, it can be concluded that differences in the test design between both the commercial and the in-house version of the competitive ELISA, i.e. using crude *B. besnoiti* tachyzoite antigens instead of semi-purified antigen and the monoclonal antibody directly conjugated to the reporter enzyme instead of using a secondary anti-mouse conjugate [11], respectively, had only a marginal effect on the inhibition values observed for tested sera in the ELISA, the commercial and the in-house Bb-ELISA1 (Fig. 2).

## Conclusions

In conclusion, the commercial version of a competitive ELISA to detect *B. besnoiti* antibodies appears to be a suitable tool to diagnose bovine besnoitiosis but needs further validation especially in cases with inconclusive, suspected false-positive or -negative results in other serological tests. In addition, a validation for use in other animal species, for example donkeys, should be considered.

## Supplementary Information

**Additional file 1: Dataset S1.** Detailed characteristics of bovine sera selected for the present study from a serum panel previously used to validate an in-house Bb-cELISA

**Additional file 2: Dataset S2.** Detailed characteristics of a set of bovine sera originating from France, The Netherlands and Norway, i.e. a set used for official validation of BIO K 466 in France.

## Data Availability

Data supporting the conclusions of this article are included within the article and its additional files. The raw datasets used and analysed during the present study are available from the corresponding author upon reasonable request.

## Abbreviations

CI: Confidence interval
ELISA: Enzyme-linked immunosorbent assay
IFAT: Immunofluorescent antibody test
